# The proteolysis targeting chimera GMB-475 combined with dasatinib for the treatment of chronic myeloid leukemia with BCR::ABL1 mutants

**DOI:** 10.3389/fphar.2022.931772

**Published:** 2022-10-03

**Authors:** Wu Ye, Xia Wu, Xiaojia Wang, Xiaoyu Wei, Yuqian Tang, Xianfeng Ouyang, Yuping Gong

**Affiliations:** Department of Hematology, West China Hospital, Sichuan University, Chengdu, Sichuan, China

**Keywords:** chronic myeloid leukemia, GMB-475, dasatinib, combination therapy, BCR::ABL1 mutants

## Abstract

Patients with chronic myeloid leukemia (CML) show resistance to tyrosine kinase inhibitors (TKIs) targeting ABL1 due to the emergence of BCR::ABL1 mutants, especially compound mutants during the treatment, which brings great challenges to clinical practice. Combination therapy is an effective strategy for drug resistance. GMB-475, a proteolysis targeting chimera (PROTAC) targeting the myristoyl pocket of ABL1 in an allosteric manner, degrades the BCR::ABL1 through the ubiquitin–proteasome pathway. In this study, we combined GMB-475 with orthosteric TKIs targeting ABL1 to overcome resistance. We constructed Ba/F3 cells carrying BCR::ABL1 mutants by gene cloning technology and compared the effects of combination therapy with those of monotherapy on the biological characteristics and signaling pathways in CML cells. We found that the effects of ABL1 inhibitors, including imatinib, dasatinib, ponatinib, and ABL001, on growth inhibition and promoting apoptosis of Ba/F3 cells with BCR::ABL1 mutants, especially compound mutants, were weakened. GMB-475 combined with TKIs, especially dasatinib, synergistically inhibited growth, promoted apoptosis, and blocked the cell cycle of Ba/F3 cells carrying BCR::ABL1 mutants and synergistically blocked multiple molecules in the JAK-STAT pathway. In conclusion, dasatinib enhanced the antitumor effect of GMB-475; that is, the combination of PROTAC targeting ABL1 in an allosteric manner and orthosteric TKIs, especially dasatinib, provides a novel idea for the treatment of CML patients with BCR::ABL1 mutants in clinical practice.

## Introduction

Chronic myeloid leukemia (CML) is a bone marrow proliferative hematopoietic cell malignancy (Eden and Coviello 2021) characterized by the *BCR::ABL1* fusion gene that is formed by genetic translocation between chromosome 9 and chromosome 22 (Haider and Anwer 2021). The BCR::ABL1 fusion oncoprotein, which has tyrosine kinase activity (Cetin et al., 2021) and activates different downstream signal pathways, such as *JAK-STAT, MAPK/ERK*, and *PI3K/Akt/mTOR*, promotes the occurrence and development of leukemia (Singh et al., 2021). In the United States, there are approximately 8000 newly diagnosed cases of CML per year (Patel et al., 2017). Imatinib, the first-generation tyrosine kinase inhibitor (TKI), significantly improved the prognosis of CML patients (Milojkovic et al., 2021; Morita and Sasaki 2021), but approximately 40% of chronic phase patients with CML had to stop imatinib due to failure and/or drug intolerance (Özgür and Eşkazan 2020; Koyama et al., 2021). The mechanisms of CML patients resistant to TKIs can be divided into BCR::ABL1-dependent and BCR::ABL1-independent resistance; the former includes BCR::ABL1 mutation and amplification, and the latter includes abnormal energy metabolism and the persistence of leukemia stem cells (Lei et al., 2021) due to bypass activation (Talati and Pinilla-Ibarz 2018). Second- and third-generation TKIs, such as dasatinib, nilotinib, bosutinib, and ponatinib, provide effective control of drug resistance caused by point mutations in the BCR::ABL1 kinase region (Liu et al., 2021), but these TKIs cannot control drug resistance caused by all site mutations. Compared with imatinib, the second-generation TKI can achieve a faster and deeper molecular response but does not prolong the survival of patients (Morita and Sasaki 2021). Moreover, some serious adverse events, such as cardiovascular toxicity of ponatinib (Singh et al., 2019) and pulmonary hypertension of dasatinib (Guignabert et al., 2016), have limited the application of these agents; meanwhile, the emergence of compound mutations in the BCR::ABL1 kinase region is resistant to all approved TKIs targeting BCR::ABL1 (Khorashad et al., 2013). ABL001, an allosteric inhibitor targeting ABL1, induces the formation of the inactive kinase conformation (Wylie et al., 2017) by binding to the myristoyl pocket of ABL1 (Breccia et al., 2021). ABL001 is effective for most single mutations in the BCR::ABL1 kinase region, but not for compound mutations, mutations in the myristoyl pocket, and the F359V mutation that affects its binding (Eide et al., 2019).

Proteolysis targeting chimera (PROTAC) has been a novel drug development technology since 2000 (Mukhamejanova et al., 2021), and it consists of three parts: ligand binding to the protein of interest, E3 ubiquitination ligase ligand (including Von Hippel–Lindau and cereblon) (Ishida and Ciulli 2021), and linker connecting the two parts (Coll-Martínez et al., 2020; Ghidini et al., 2021). PPOTAC, binding to the target protein and recruiting E3 ubiquitination ligase, ubiquitinates the target protein and then degrades it by the proteasome, which achieves an antitumor effect (Qi et al., 2021). Being widely applied as a biological tool and drug molecule, PROTAC has a potential clinical application value (Zeng et al., 2021) and is considered a novel strategy for the treatment of various diseases (Mukhamejanova et al., 2021). At present, many PROTAC molecules with high degradation efficiency have been reported, including those targeting the androgen receptor (Lee et al., 2021), estrogen receptor (Jiang et al., 2018; Hu et al., 2019; Li et al., 2019), ALK (Yan et al., 2021), BTK (Buhimschi et al., 2018; Sun et al., 2018; Zhao et al., 2021), and many others. The first batch of oral PROTACs has been included in clinical trials achieving exciting results (Protein Degradation, 2020; Qin et al., 2021). Unlike traditional small molecule inhibitors needing stable binding with the target protein, as long as PROTAC binds to the target protein briefly, it would degrade it in a catalytic manner (Martín-Acosta and Xiao 2021). GMB-475 (Burslem et al., 2019), targeting the myristoyl pocket of ABL1 via an allosteric way, degrades the BCR::ABL1 fusion protein through the ubiquitin–proteasome pathway; however, it inhibited proliferation and promoted apoptosis in CML cell lines only at high concentrations. Thus, we combined GMB-475 with orthosteric TKIs targeting ABL1 to reduce the effective concentration of the two drugs. Therefore, this study investigated the combination of GMB-475 and TKIs to overcome drug resistance in CML caused by BCR::ABL1 mutations.

## Materials and methods

The sources of the main experimental reagents are shown in Supplementary Table S1.

### Cell lines

Using the MSCV-IRES-GFP-p210 (MIG-p210) wild-type plasmid as a template, we constructed MIG-p210 plasmids with BCR::ABL1 single mutations (including E255K, T315I, L387M, F359V, and F486S) by PCR, overlapping PCR, enzyme digestion and enzyme connection, gel purification, and recovery. Then, using MIG-p210 plasmids with BCR::ABL1 single mutations as a template and repeating the above process, we constructed MIG-p210 plasmids with BCR::ABL1 compound mutations (including T315I + E255K, T315I + L387M, and T315I + F486S). After sequencing the MIG-p210 plasmids with BCR:ABL1 mutations, we confirmed that the mutations were successfully introduced at the expected design sites. We obtained MIG-p210 retrovirus that was used to infect Ba/F3 cells via calcium phosphate and then screened Ba/F3 cells stably expressing BCR-ABL1 mutants (Ba/F3-MIG-p210). The cell lines used in this study are shown in Table 1.

**TABLE 1:** The source of cell lines and the required culture medium.

Cell lines	Sources	Culture medium
Ba/F3	Institute of Hematology, West China Hospital	RPMI 1640 (1 ng/ml IL3)
K562	Institute of Hematology, West China Hospital	RPMI 1640
Ba/F3-MIG-p210^WT^	This study	RPMI 1640
Ba/F3-MIG-p210^E255K^	This study	RPMI 1640
Ba/F3-MIG-p210^T315I^	This study	RPMI 1640
Ba/F3-MIG-p210^F359V^	This study	RPMI 1640
Ba/F3-MIG-p210^L387M^	This study	RPMI 1640
Ba/F3-MIG-p210^F486S^	This study	RPMI 1640
Ba/F3-MIG-p210^T315I+E255K^	This study	RPMI 1640
Ba/F3-MIG-p210^T315I+L387M^	This study	RPMI 1640
Ba/F3-MIG-p210^T315I+F486S^	This study	RPMI 1640
Ba/F3-MIG- p210-Luc	This study	RPMI 1640

Ba/F3-MIG-p210 cells: Ba/F3 cells were transfected with MSCV-IRES-GFP-P210 (MIG-P210) retrovirus to make them express BCR::ABL1 fusion protein with the molecular weight of 210 kDa.

### Cell viability analysis

We inoculated Ba/F3-MIG-p210 cells into 96-well plates with 8000 or 3000 cells per well and added different concentrations of TKIs (0–2 µM) (to better distinguish the different effects of ABL1 inhibitors against BCR::ABL1 compound mutations, single-point mutation, and WT, we used drug concentrations less than 2 µM), ABL001 (0–5 µM), or GMB-475 (0–5 µM) (to observe the therapeutic effect of GMB-475 on mutant cells, the maximum concentration was increased to 5 µM); the total volume of each well was 100 µl, and the cells were placed in an incubator at 37 °C and 5% CO_2_ for 24/48 h. There were two ways to detect cell viability afterward: 1) After adding 20 µl of 5 mg/ml thiazolyl blue tetrazolium bromide (MTT) per well and waiting for 4–6 h, we added 100 µl of MTT-dissolved solution per well and dissolved it in an incubator at 37°C overnight and measured the absorbance value of each well at 570 nm with a spectrometer the next day; 2) after adding 10 µl of Cell Counting Kit-8 (CCK8) per well and waiting for 3 h, we detected the absorbance value of each well at 450 nm with a spectrometer. The curve of cell viability was drawn with GraphPad Prism 8.0, and CompuSyn software was used to calculate the drug combination index (CI) of GMB-475 with dasatinib or ponatinib. A CI value less than 1 indicates a synergistic effect (a smaller CI indicates a better synergistic effect), while a CI value equal to 1 indicates an additive effect, and a CI value greater than 1 indicates an antagonistic effect.

### Cell apoptosis

We inoculated Ba/F3-MIG-p210 cells into six-well plates with 1×10^5^ cells per well and added different concentrations of TKIs (0–2 µM), ABL001 (0–5 µM), or GMB-475 (0–5 µM); the total volume of each well was 2 ml, and the cells were placed in an incubator at 37 °C and 5% CO_2_ for 24–48 h. Cell apoptosis was detected by annexin V-647 and 7-aminoactinomycin D (7-AAD) double staining. The results were statistically analyzed by GraphPad Prism 8.0 software.

### CML mouse model study

Ba/F3-MIG-p210 cells were transfected with HBLV-luciferase-blasticidin virus to construct a cell line expressing luciferase (Ba/F3-MIG-p210-Luc cell line). Four to 6 hours after 3.8 Gy X-ray irradiation, each 8-week-old Balb/c mouse was injected with 3 × 10^5^ Ba/F3-MIG-p210-Luc cells via the tail vein. The mice were administered intraperitoneally with the drug after 72 h. The experimental group was treated with 5 mg/kg GMB-475 once every 2 days (from days 4 to 14), and the control group was injected with the corresponding volume of drug solvent (4% DMSO + 30% PEG300 + 5% Tween 80 + ddH_2_O). There were 12 mice in the experimental group and the control group, with 6 mice in each group. The tumor burden of the mice was observed by luminescence imaging, and the survival of the mice was recorded throughout the process.

### Cell cycle

We inoculated Ba/F3-MIG-p210 cells into six-well plates with 2×10^5^ cells per well and added different concentrations of dasatinib (1 µM or 2 µM) or GMB-475 (1 µM or 2 µM) (to avoid too many dead cells during the experiment, we used the drug concentration lower than or equal to 2 µM); the total volume of each well was 2 ml, and the cells were placed in an incubator at 37°C and 5% CO_2_ for 48 h. The cells were collected and fixed with 70% ethanol solution overnight, digested with RNase, and stained with propidium iodide (PI). The cell cycle was detected using flow cytometry and analyzed with Modfit software.

### Real-time fluorescence quantitative PCR

The changes of mRNA levels induced by the corresponding drugs were detected by real-time fluorescence quantitative PCR (qPCR), and the primer sequences of the genes used for qPCR are shown in Supplementary Table S2.

### Western blot

We inoculated Ba/F3-MIG-p210 cells into 6-cm Petri dishes and added different concentrations of drugs; the total volume of each dish was 5 ml, and the cells were placed in an incubator at 37°C and 5% CO_2_ for 24/48 h. Then, the proteins of cells were extracted, and the levels of BCR::ABL1 protein and related signal pathway proteins were detected by Western blot.

### Statistical analysis

We adopted Kaplan–Meier analysis and the log-rank test to compare the survival of the CML mouse model between the experimental group and the control group. In the Cell Experiments section, the results represent the mean ± standard error of two or three independent experiments. The unpaired *t*-test was used to compare the differences between the two groups, and *p* values less than 0.05 showed significant differences.

## Results

### The effects of inhibitors targeting ABL1 on the growth inhibition of Ba/F3-MIG-p210 cells with BCR::ABL1 compound mutations were significantly weakened

**FIGURE 1:**
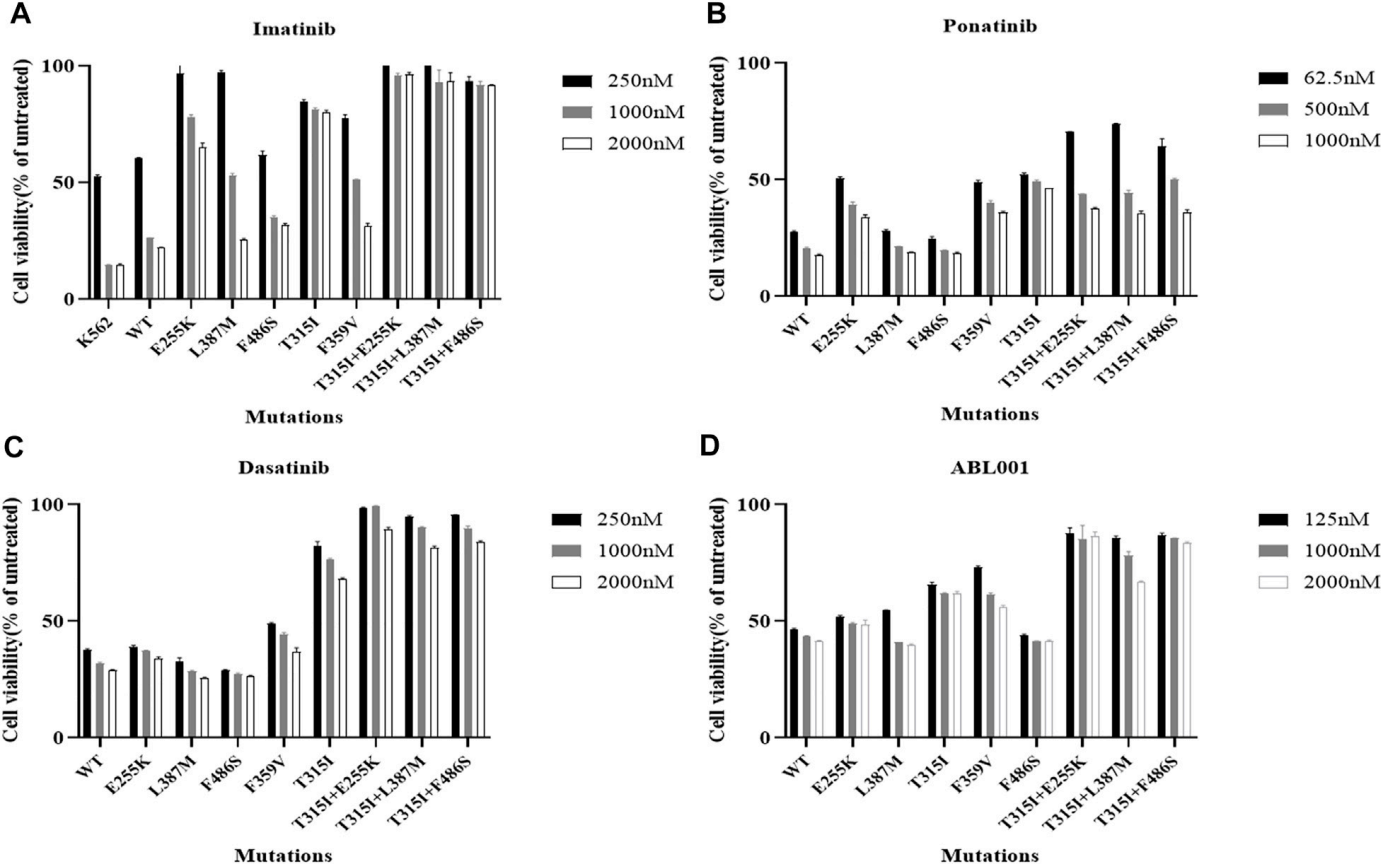
Effects of inhibitors targeting ABL1 on the growth inhibition of Ba/F3-MIG-p210 cells with BCR::ABL1 compound mutations were significantly weakened. The viability of Ba/F3-MIG-p210 cells treated with different concentrations of ABL1 inhibitor for 48 h was detected via the MTT assay: **(A)** imatinib, **(B)** ponatinib, **(C)** dasatinib, and **(D)** ABL001.

The viability of Ba/F3-MIG-p210 cells treated with different concentrations of ABL1 inhibitor for 48 h was detected via the MTT assay. The results showed that Ba/F3-MIG-p210 cells with different BCR::ABL1 mutations were generally resistant to imatinib, especially compound mutations, and imatinib did not inhibit cell growth when the concentration reached 2000 nM (Figure 1A). In contrast, dasatinib significantly inhibited the growth of Ba/F3-MIG-p210 cells with the BCR::ABL1 single-point mutation but weakly inhibited the growth of those with compound mutations (Figure 1C). Ponatinib and ABL001 also performed more effectively on Ba/F3-MIG-p210 cells with BCR::ABL1 single-point mutations than those with compound mutations in terms of growth inhibition (Figures 1B,D).

### The effects of ABL1 inhibitors on promoting apoptosis of Ba/F3-MIG-p210 cells with BCR::ABL1 mutants were weakened

**FIGURE 2:**
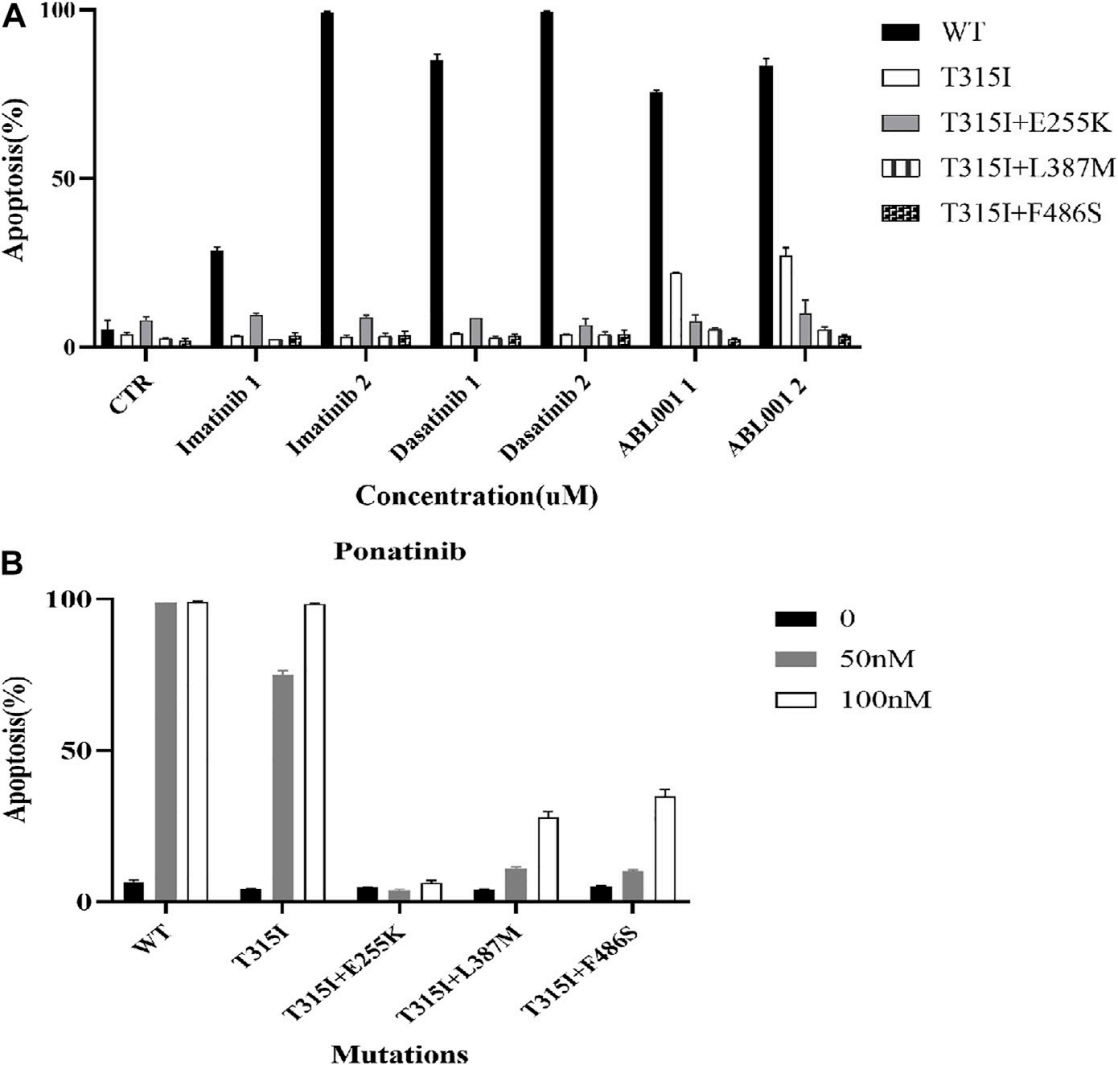
Effects of ABL1 inhibitors on promoting apoptosis of Ba/F3-MIG-p210 cells with BCR::ABL1 mutants were weakened. The apoptosis of Ba/F3-MIG-p210 cells treated with different ABL1 inhibitors for 48 h was detected via annexin V-647 and 7-AAD double staining: **(A)** Imatinib, dasatinib, and ABL001; the abscissa marks the names and concentrations of agents, and the ordinate is the apoptosis rate; the colors of the bar graph represent the BCR::ABL1^wild type^ (WT) or different mutants carried by Ba/F3-MIG-p210 cells, including BCR::ABL1^T315I^, BCR::ABL1^T315I+E255K^, BCR::ABL1^T315I+L387M^, and BCR::ABL1^T315I+F486S^. **(B)** Ponatinib; the abscissa marks the BCR::ABL1^WT^, or different mutants carried by Ba/F3-MIG-p210 cells, and the ordinate is the apoptosis rate; the colors of the bar graph represent different concentrations of ponatinib.

The apoptosis of Ba/F3-MIG-p210 cells treated with different ABL1 inhibitors for 48 h was detected via annexin V-647 and 7-AAD double staining. The results showed that compared with BCR::ABL1 wild-type, the effects of imatinib, dasatinib, and ABL001 on promoting apoptosis in Ba/F3-MIG-p210 cells with T315I or T315I-including compound mutations, were significantly reduced under the same concentrations and treatment times of agents (Figure 2A). Compared with BCR::ABL1 wild-type or T315I single-point mutation, the effects of promoting apoptosis of ponatinib on cells with BCR::ABL1 compound mutations were also significantly weakened (Figure 2B).

### GMB-475 exhibited a growth inhibition effect on Ba/F3-MIG-p210 cells with BCR::ABL1^T315I+F486S^ mutations but no significant improvement of prognosis in chronic myeloid leukemia mouse models constructed by this cell line

**FIGURE 3:**
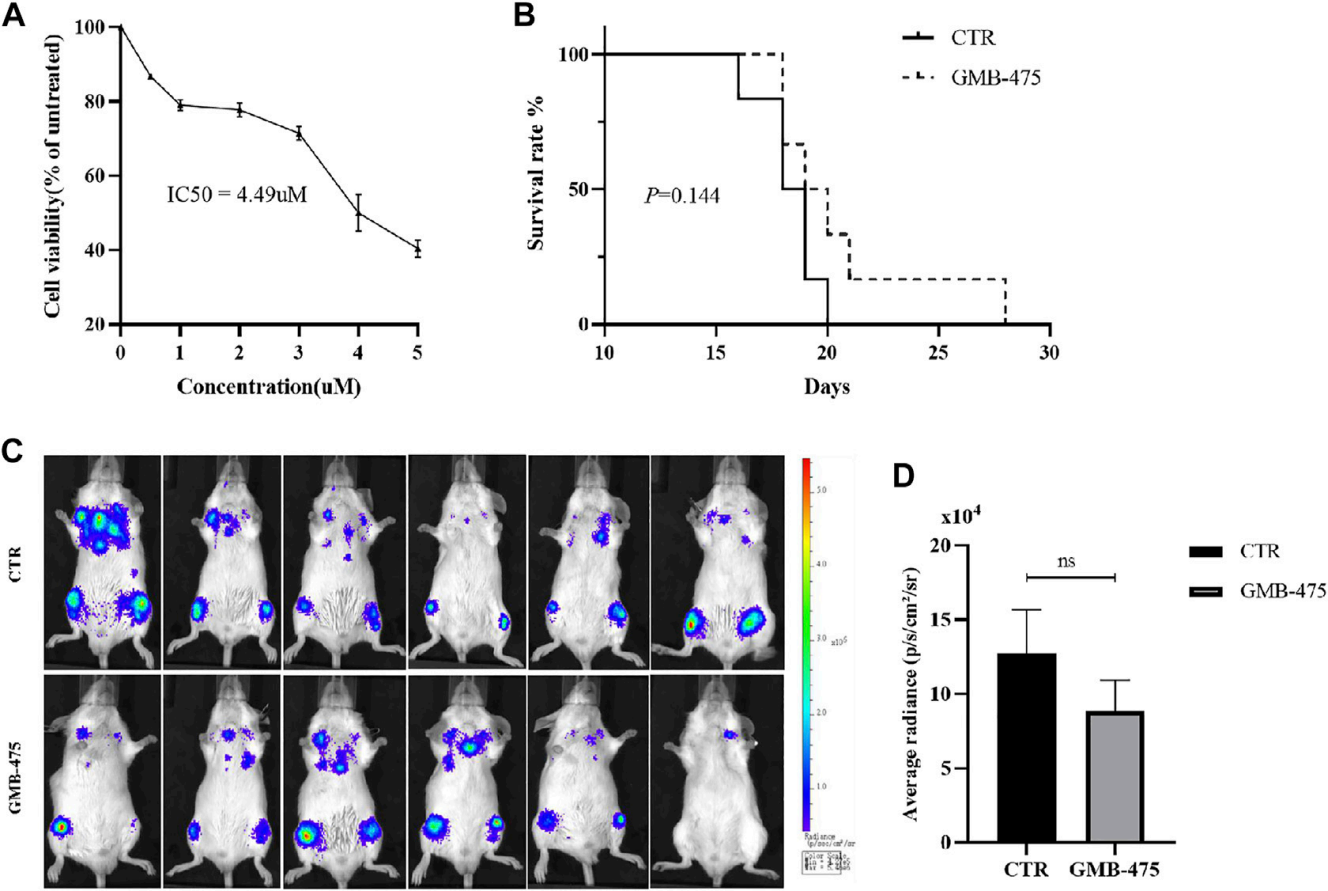
GMB-475 exhibited a growth inhibition effect on Ba/F3-MIG-p210 cells carrying BCR::ABL1^T315I+F486S^ mutations but no significant improvement of prognosis in the CML mouse model constructed by this cell line. **(A)** Ba/F3-MIG-p210 cells carrying BCR::ABL1^T315I+F486S^ mutations were treated with different concentrations of GMB-475 for 48 h, and cell viability was detected using the CCK8 assay. **(B)** Survival curve of the CML mouse model for the control group and the GMB-475 administration group. **(C)** Fluorescence imaging of CML mouse model was implemented at day 9. **(D)** Quantitative analysis of the fluorescence signal was performed to measure the tumor burden in the CML mouse model. Abbreviations: CTR, control.

Ba/F3-MIG-p210 cells carrying BCR::ABL1^T315I+F486S^ mutations were treated with different concentrations of GMB-475 for 48 h, and cell viability was detected using the CCK8 assay. The results showed that GMB-475 exhibited a growth inhibition effect on Ba/F3-MIG-p210 cells carrying BCR::ABL1^T315I+F486S^ mutations, and the half inhibitory concentration (IC50) was 4.49 µM (Figure 3A). Twelve 8-week-old Balb/c mice were randomly divided into two groups with six mice in each group: the control group and the GMB-475 administration group. Four to 6 hours after 3.8 Gy X-ray irradiation, each mouse was injected with 3 × 10^5^ Ba/F3-MIG-p210-Luc cells carrying BCR::ABL1^T315I+F486S^ mutations via the tail vein. The mice were administered drugs intraperitoneally after 72 h. The experimental group was treated with 5 mg/kg GMB-475 once every 2 days (from days 4 to 14), and the control group was injected with the corresponding volume of drug solvent. The tumor burden of mice was observed by luminescence imaging at day 9, and the survival of mice was recorded throughout the process. The results showed that although GMB-475 showed a trend of reducing the tumor burden (Figure 3C,D) and prolonging the survival of the CML mouse model (Figure 3B), its effect was limited and not statistically significant. In view of the results, we considered the combination of GMB-475 and TKIs to improve the antitumor effect.

### GMB-475 combined with tyrosine kinase inhibitors showed synergistic effects of growth inhibition on Ba/F3-MIG-p210 cells with BCR::ABL1 mutants

**FIGURE 4:**
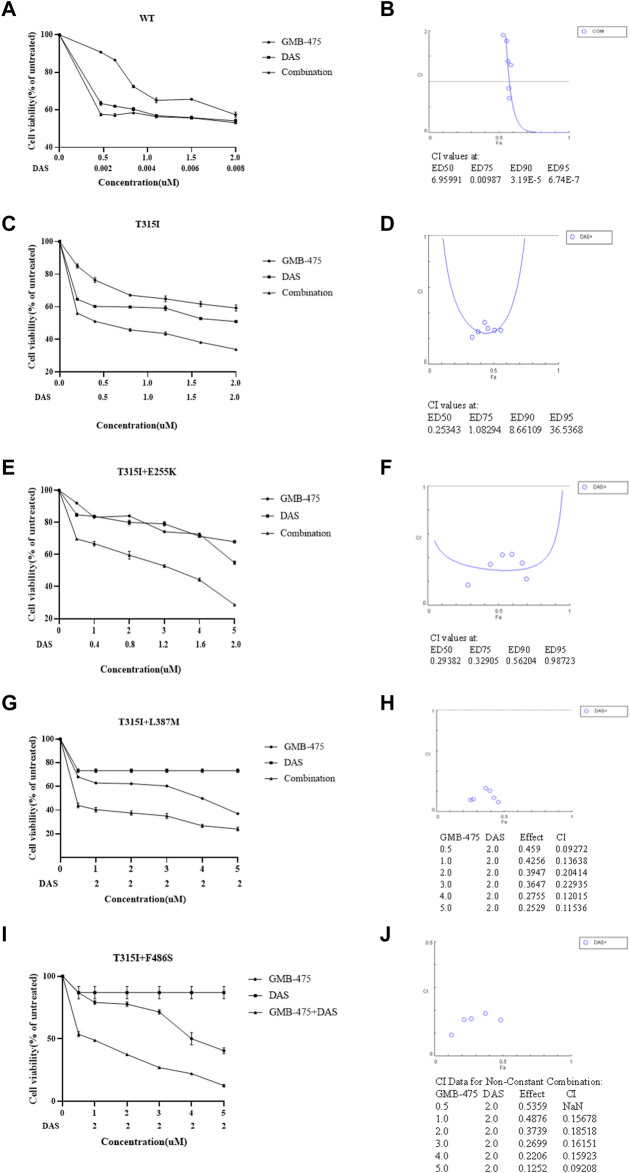
GMB-475 combined with dasatinib (DAS) had synergistic effects on the growth inhibition of Ba/F3-MIG-p210 cells with BCR::ABL1 mutants but no synergistic effect on that with BCR::ABL1^WT^. **(A,C,E,G,I)** The viability of Ba/F3-MIG-p210 cells carrying BCR::ABL1^WT^, BCR::ABL1^T315I^, BCR::ABL1^T315I+E255K^, BCR::ABL1^T315I+L387M^, or BCR::ABL1 ^T315I+F486S^ mutations treated with different concentrations of GMB-475, dasatinib, or GMB-475 plus dasatinib for 48 h was detected using the CCK8 assay. The abscissa represents the concentrations of GMB-475, and the corresponding concentrations of dasatinib are marked below the abscissa; the ordinate is the cell survival rate. **(B,D,F,H,G)** The curve figures of combination indexes (CIs); the CIs of GMB-475 combined with dasatinib at ED50, ED75, ED90, and ED95, or the CIs for different concentrations of GMB-475 combined with 2-µM dasatinib are shown below the figures; the CI at ED50 was the overall CI.

The distinct effects of growth inhibition between GMB-475 combined with TKIs and single agents on Ba/F3-MIG-p210 cells were detected using the CCK8 assay. We found that the overall combination index (CI) of GMB-475 and dasatinib in Ba/F3-MIG-p210 cells with BCR::ABL1^WT^ was 6.96, and the two drugs showed no synergistic effect of growth inhibition on cells (Figures 4A,B). In Ba/F3-MIG-p210 cells with BCR::ABL1^T315I^ or BCR::ABL1^T315I+E255K^ mutations, the overall CIs of GMB-475 and dasatinib were 0.25 and 0.29, respectively, and the two drugs exhibited significant synergistic effects of growth inhibition on cells (Figures 4C–F). When the concentration of dasatinib was fixed at 2 µM and GMB-475 was set at different concentrations, the combination of the two drugs also showed significant synergistic effects of growth inhibition on Ba/F3-MIG-p210 cells with BCR::ABL1^T315I+L387M^ or BCR::ABL1^T315I+F486S^ mutations (the CIs of each concentration were less than 0.54, Figures 4G–J). Meanwhile, dasatinib significantly reduced the IC50 of GMB-475 in Ba/F3-MIG-p210 cells with BCR::ABL1^T315I^, BCR::ABL1^T315I+E255K^, BCR::ABL1^T315I+L387M^, and BCR::ABL1^T315I+F486S^ mutations (Table 2). Moreover, we also found that GMB-475 combined with ponatinib also showed synergistic effects of growth inhibition on Ba/F3-MIG-p210 with BCR::ABL1^T315I+E255K^ or BCR::ABL1^T315I+F486S^ mutations; the overall CIs were 0.67 and 0.61, and the highest CIs at different concentrations were 0.98 and 1.02, respectively (Supplementary Figures S1A–D). The synergistic effect of GMB-475 combined with ponatinib was weaker than that with dasatinib.

**TABLE 2:** The IC50 of GMB-475 in Ba/F3 cells with BCR::ABL1 mutants.

	IC50 of GMB-475 (µM)	IC50 of GMB-475^δ^ combined with dasatinib (µM)
Ba/F3-MIG-p210^T315I^	3.69	0.44
Ba/F3-MIG-p210^T315I+E255K^	8.29	2.57
Ba/F3-MIG-p210^T315I+L387M^	3.70	0.31
Ba/F3-MIG-p210^T315I+F486S^	4.49	0.77

^δ^ The IC50 of GMB-475 against Ba/F3-MIG-p210 cells when GMB-475 combined with dasatinib.

### GMB-475 combined with tyrosine kinase inhibitors synergistically promoted the apoptosis of Ba/F3-MIG-p210 cells

**FIGURE 5:**
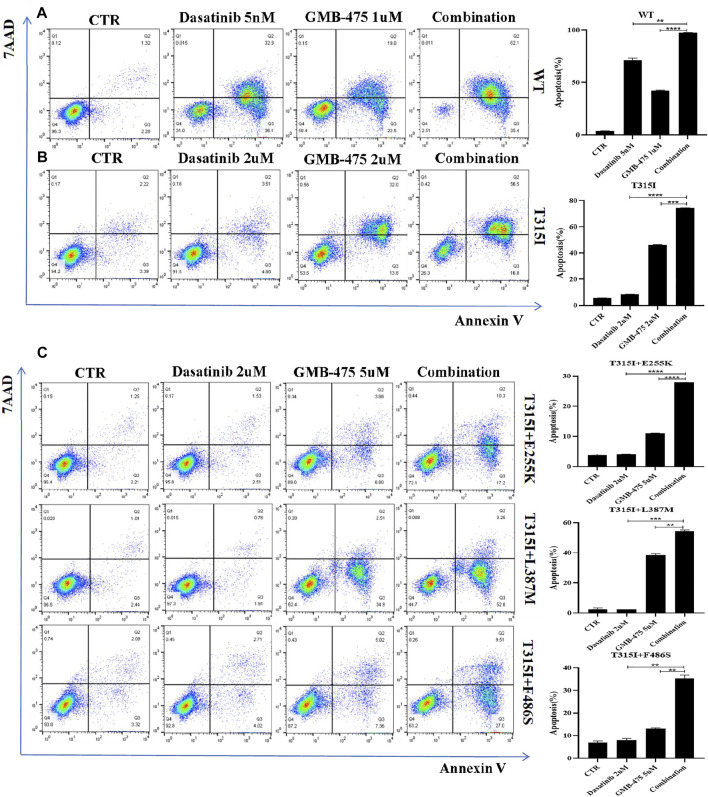
GMB-475 combined with dasatinib synergistically promoted the apoptosis of Ba/F3-MIG-p210 cells. The apoptosis of Ba/F3-MIG-p210 cells treated with control medium (CTR), GMB-475, dasatinib, or GMB-475 plus dasatinib for 48 h was detected via annexin V and 7-AAD double staining: **(A)** Ba/F3-MIG-p210 cells with BCR::ABL1^WT^ were treated with CTR, dasatinib 5nM, GMB-475 1 µM, and GMB-475 1 µM plus dasatinib 5 nM. **(B)** Ba/F3-MIG-p210 cells carrying BCR::ABL1^T315I^ were treated with CTR, dasatinib 2 µM, GMB-475 2 µM, and GMB-475 2 µM plus dasatinib 2 µM. **(C)** Ba/F3-MIG-p210 cells carrying BCR::ABL1^T315I+E255K^, BCR::ABL1^T315I+L387M^, or BCR::ABL1 ^T315I+F486S^ compound mutations were treated with CTR, dasatinib 2 µM, GMB-475 5 µM, and GMB-475 5 µM plus dasatinib 2 µM, respectively. **p < 0.01, ***p < 0.001, ****p < 0.0001.

The apoptosis of Ba/F3-MIG-p210 cells with BCR::ABL1 wild-type or mutants induced by GMB-475 combined with TKIs was detected by flow cytometry. The results showed that GMB-475 combined with dasatinib synergistically promoted the apoptosis of Ba/F3-MIG-p210 cells (Figure 5). Meanwhile, GMB-475 combined with ponatinib synergistically promoted the apoptosis of Ba/F3-MIG-p210 cells with BCR::ABL1^T315I+E255K^ and BCR::ABL1^T315I+F486S^ mutations (Supplementary Figure S2).

### GMB-475 combined with dasatinib exhibited a better synergistic effect on Ba/F3-MIG-p210 cells carrying BCR::ABL1^T315I+F486S^ mutations compared with ABL001

**FIGURE 6:**
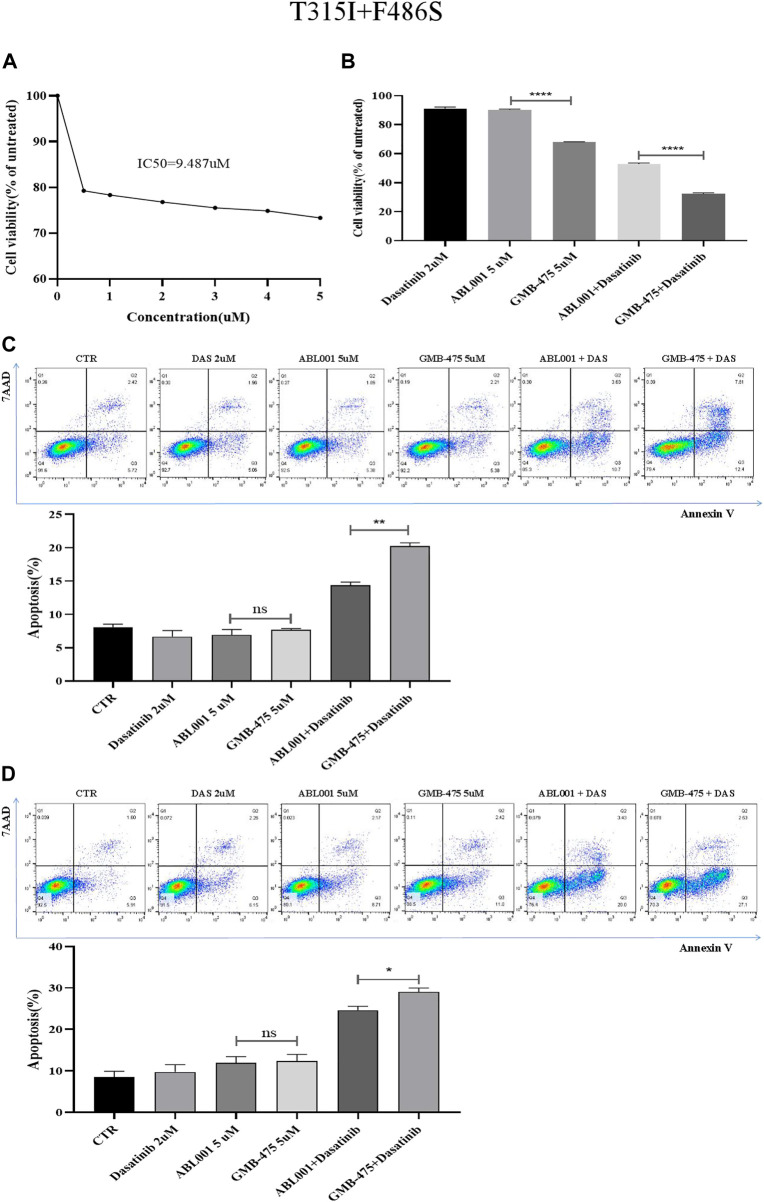
GMB-475 combined with dasatinib exhibited a better synergistic effect on Ba/F3-MIG-p210 cells carrying BCR::ABL1^T315I+F486S^ mutations compared with ABL001. **(A)** Ba/F3-MIG-p210 cells were treated with different concentrations of ABL001 for 48 h, and cell viability was detected using the CCK8 assay. **(B)** The viability of Ba/F3-MIG-p210 cells treated with dasatinib 2 µM, ABL001 5 µM, GMB-475 5 µM, ABL001 5 µM plus dasatinib 2 µM, and GMB-475 5 µM plus dasatinib 2 µM for 24 h was detected using the CCK8 assay. **(C)** The apoptosis of Ba/F3-MIG-p210 cells treated with control medium (CTR), dasatinib 2 µM, ABL001 5 µM, GMB-475 5 µM, ABL001 5 µM plus dasatinib 2 µM, and GMB-475 5 µM plus dasatinib 2 µM for 24 h was detected via annexin V and 7-AAD double staining. **(D)** Ba/F3-MIG-p210 cells were treated with CTR, dasatinib 2 µM, ABL001 5 µM, GMB-475 5 µM, ABL001 5 µM plus dasatinib 2 µM, and GMB-475 5 µM plus dasatinib 2 µM for 24 h and continued to be cultured in complete medium without drugs for 18 h; then the apoptosis of those cells was detected. ^ns^ *p ≥* 0.05, *p < 0.05, **p < 0.01, ****p < 0.0001.

Ba/F3-MIG-p210 cells were treated with different concentrations of ABL001 for 48 h, and cell viability was detected using the CCK8 assay. The IC50 of ABL001 was 9.487 µM (Figure 6A); in contrast, the IC50 of GMB-475 was 4.49 µM. The viability of Ba/F3-MIG-p210 cells treated with different agents for 24 h was detected using the CCK8 assay. The results showed that GMB-475 alone or in combination with dasatinib showed more significant growth inhibition on Ba/F3-MIG-p210 cells carrying BCR::ABL1^T315I+F486S^ mutations than ABL001 alone or in combination with dasatinib, respectively (Figure 6B). The apoptosis of Ba/F3-MIG-p210 cells treated with different agents for 24 h was detected by annexin V and 7-AAD double staining. The results showed that there was no difference between GMB-475 and ABL001 in promoting apoptosis of cells; however, the apoptosis rate of cells treated with GMB-475 combined with dasatinib was significantly higher than that treated with ABL001 combined with dasatinib (Figure 6C). Ba/F3-MIG-p210 cells were treated with different agents for 24 h and continued to be cultured in a complete medium without drugs for 18 h; then the apoptosis of those cells was detected. The results showed that there was no difference between GMB-475 and ABL001 in promoting apoptosis of cells, and the apoptosis rate of cells treated with GMB-475 combined with dasatinib was higher than that treated with ABL001 combined with dasatinib (Figure 6D). GMB-475 combined with dasatinib exhibited a better synergistic effect compared with ABL001 combined with dasatinib.

### GMB-475 combined with dasatinib synergistically blocked the cell cycle of Ba/F3-MIG-p210 cells with BCR::ABL1 mutants

**FIGURE 7:**
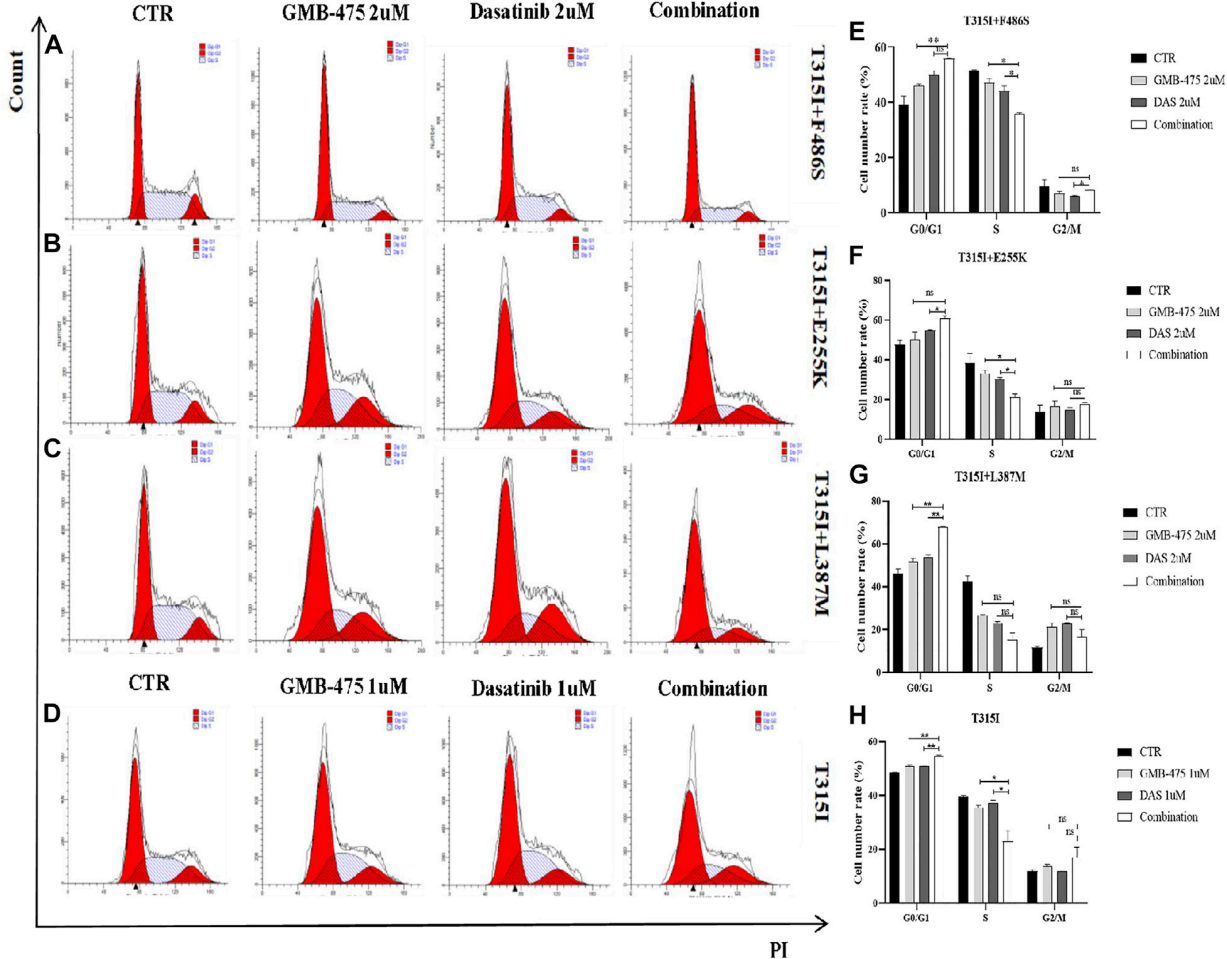
GMB-475 combined with dasatinib synergistically blocked the cell cycle of Ba/F3-MIG-p210 cells with BCR::ABL1 mutants. The cell cycle of Ba/F3-MIG-p210 cells treated with the control medium (CTR), GMB-475, dasatinib (DAS), or GMB-475 plus dasatinib for 48 h was detected by PI staining: **(A–C)** Ba/F3-MIG-p210 cells carrying BCR::ABL1^T315I+F486S^, BCR::ABL1^T315I+E255K^, or BCR::ABL1^T315I+L387M^ compound mutations were treated with CTR, dasatinib 2 µM, GMB-475 2 µM, and GMB-475 2 µM plus dasatinib 2 µM, respectively. **(D)** Ba/F3-MIG-p210 cells carrying BCR::ABL1^T315I^ were treated with CTR, dasatinib 1 µM, GMB-475 1 µM, and GMB-475 1 µM plus dasatinib 1 µM. **(E–H)** Statistical analysis results of the cell cycle. ^ns^ *p* ≥ 0.05, *p < 0.05, **p < 0.01.

The effects of blocking the cell cycle for GMB-475 combined with dasatinib on Ba/F3-MIG-p210 cells with BCR::ABL1 mutants were detected by PI staining. The results showed that compared with single agents, the proportion of cells in the G0/G1 phase increased but that in the S phase (DNA synthesis phase) decreased under combination therapy; in other words, the effect of blocking the cell cycle of combination therapy was more obvious than that of single agents (Figure 7).

### GMB-475 combined with dasatinib synergistically downregulated the mRNA levels of the *JAK-STAT* axis, *AKT*, and *mTOR* in Ba/F3-MIG-p210 cells with BCR::ABL1 mutants

**FIGURE 8:**
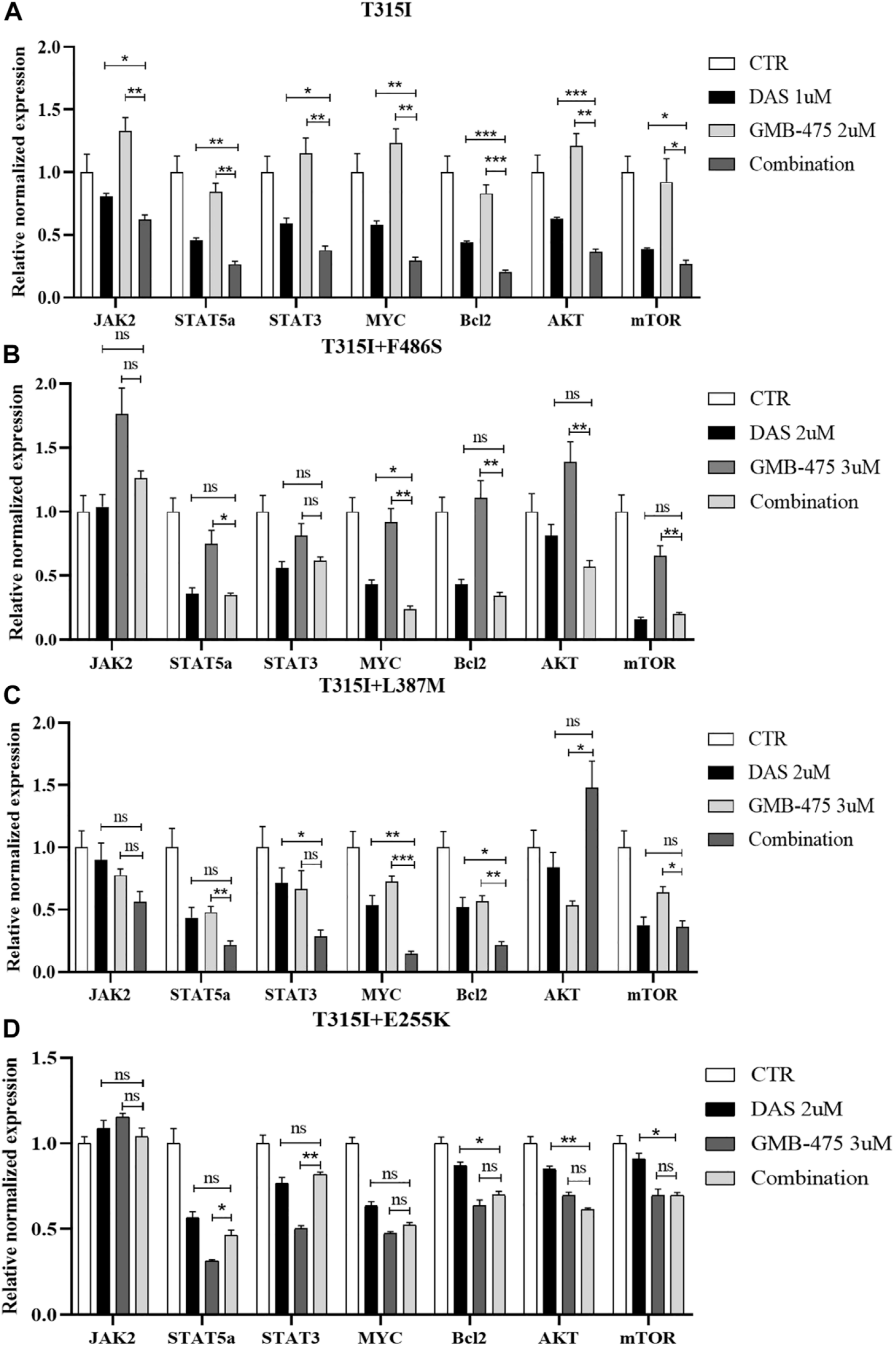
GMB-475 combined with dasatinib synergistically downregulated the mRNA levels of the *JAK-STAT* axis, *AKT*, and *mTOR* in Ba/F3-MIG-p210 cells with BCR::ABL1 mutants. The mRNA levels of genes in Ba/F3-MIG-p210 cells treated with control medium (CTR), GMB-475, dasatinib (DAS), or GMB-475 plus dasatinib for 48 h were detected by qPCR: **(A)** Ba/F3-MIG-p210 cells carrying BCR::ABL1^T315I^ mutation were treated with CTR, dasatinib 1 µM, GMB-475 2 µM, and GMB-475 2 µM plus dasatinib 1 µM. **(B–D)** Ba/F3-MIG-p210 cells carrying BCR::ABL1^T315I+F486S^, BCR::ABL1^T315I+L387M^, or BCR::ABL1^T315I+E255K^ compound mutations were treated with CTR, dasatinib 2µM, GMB-475 3 µM, and GMB-475 3 µM plus dasatinib 2 µM, respectively. The types of BCR::ABL1 mutants carried by Ba/F3-MIG-p210 cells are marked at the top of the figures. ^ns^ *p* ≥ 0.05, *p < 0.05, **p < 0.01, ***p < 0.001.

The distinct mRNA levels of genes induced by GMB-475 combined with dasatinib and single agents were detected by qPCR. Compared with the BCR::ABL1^T315I^ single-point mutation, Ba/F3-MIG-p210 cells carrying compound mutations are more resistant to the agents. To observe the change in mRNA levels, the concentrations of the agents were increased when we treated Ba/F3-MIG-p210 cells carrying compound mutations. The results showed that combination therapy downregulated the expression of the *JAK-STAT* pathway in cells compared with the single drugs. In Ba/F3-MIG-p210 cells with the BCR::ABL1^T315I^ mutation, the mRNA levels of *JAK2*, *STAT5a*, *STAT3*, *MYC*, and *BCL2* of the *JAK-STAT* pathway, and *AKT* and *mTOR* that are downstream genes of *JAK*, were downregulated (Figure 8A). The mRNA levels of *MYC* and *MYC* plus *BCL2* were also significantly downregulated in the two Ba/F3-MIG-p210 cell lines with BCR::ABL1^T315I+F486S^ and BCR::ABL1^T315I+L387M^ mutations, respectively. The changes in the mRNA levels of other genes were not statistically significant under the induction of combination therapy, including *JAK2*, *STAT5a*, *STAT3*, *AKT*, and *mTOR* (Figures 8B,C). However, in Ba/F3-MIG-p210 cells with BCR::ABL1^T315I+E255K^ compound mutations, there was no significant distinction in the mRNA levels of genes induced by combination therapy and single drugs (Figure 8D).

### GMB-475 combined with dasatinib synergistically downregulated the levels of some proteins in the JAK-STAT axis or AKT in Ba/F3-MIG-p210 cells with BCR::ABL1 mutants

**FIGURE 9:**
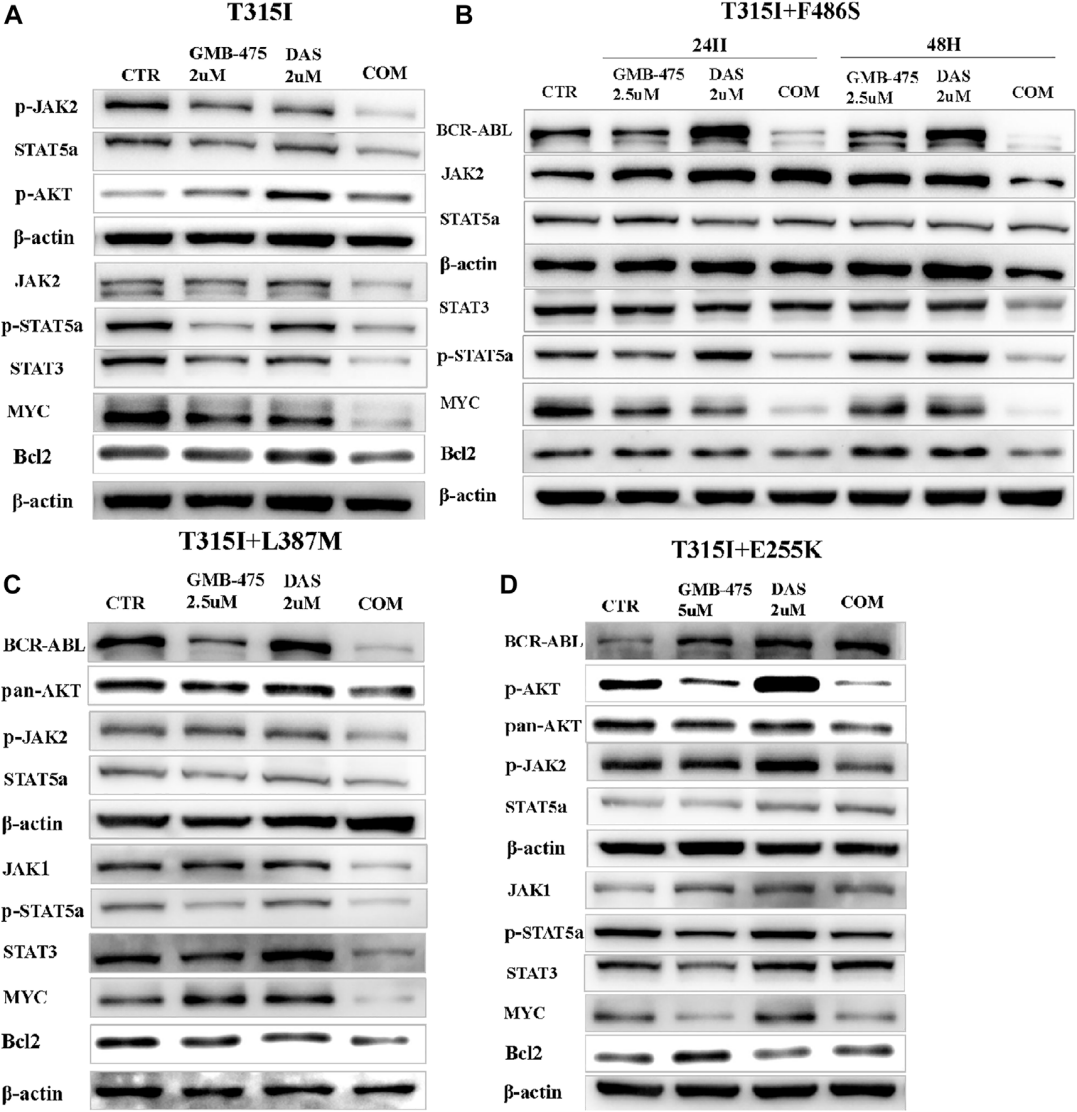
GMB-475 combined with dasatinib synergistically downregulated the protein levels of the JAK-STAT axis or AKT in Ba/F3-MIG-p210 cells with BCR::ABL1 mutants. The protein levels of genes in Ba/F3-MIG-p210 cells treated with the control medium (CTR), GMB-475, dasatinib (DAS), or GMB-475 plus dasatinib were detected by western blot: **(A)** Ba/F3-MIG-p210 cells carrying BCR::ABL1^T315I^ were treated with CTR, GMB-475 2 µM, dasatinib 2 µM, and GMB-475 2 µM plus dasatinib 2 µM for 48 h. **(B,C)** Ba/F3-MIG-p210 cells carrying BCR::ABL1 ^T315I+F486S^ and BCR::ABL1^T315I+L387M^ compound mutations were treated with CTR, GMB-475 2.5 µM, dasatinib 2 µM, and GMB-475 2.5 µM plus dasatinib 2 µM for 48 h, respectively (Ba/F3-MIG-p210 cells with BCR::ABL1^T315I+F486S^ were treated for 24 and 48 h). **(D)** Ba/F3-MIG-p210 cells carrying BCR::ABL1^T315I+E255K^ were treated with CTR, GMB-475 5 µM, dasatinib 2 µM, and GMB-475 5 µM plus dasatinib 2 µM for 48 h. The types of BCR::ABL1 mutants carried by Ba/F3-MIG-p210 cells are marked at the top of the figures.

The levels of protein expression of genes in cells induced by agents were detected by WB. The results showed that GMB-475 combined with dasatinib downregulated the protein levels of the JAK-STAT pathway in cells compared with single agents. In Ba/F3-MIG-p210 cells with the BCR::ABL1^T315I^ mutation, the levels of JAK2, p-JAK2, STAT5a, STAT3, and MYC decreased significantly under combination therapy compared with single agents (Figure 9A). In Ba/F3-MIG-p210 cells with BCR::ABL1^T315I+F486S^ compound mutations, the levels of BCR::ABL1, p-STAT5a, STAT3, MYC, and Bcl2 decreased significantly, but JAK2 increased at 24 h under combination therapy compared with single agents (Figure 9B). Similarly, the levels of BCR::ABL1, JAK1, p-JAK2, p-STAT5a, STAT3, MYC, and Bcl2 decreased in Ba/F3-MIG-p210 cells with BCR::ABL1^T315I+L387M^ compound mutations under combination therapy compared with single agents (Figure 9C). However, in Ba/F3-MIG-p210 cells with BCR::ABL1^T315I+E255K^ compound mutations, the levels of BCR::ABL1, STAT3, STAT5, and p-STAT5a were upregulated, MYC and Bcl2 remained unchanged, but p-JAK2, pan AKT, and p-AKT decreased under combination therapy compared with single agents (Figure 9D).

## Discussion

CML is characterized by the BCR::ABL1 fusion gene, which is formed by a genetic translocation between chromosome 9 and chromosome 22 (Rinke et al., 2020). ABL1 is a proto oncogene encoding tyrosine kinase protein that is involved in a variety of cellular processes in humans, including cell division, adhesion, differentiation, and stress response. Normally, the tyrosine kinase protein encoded by ABL1 is negatively regulated by its N-terminal myristoyl peptide (Radi et al., 2013), the sequence encoding which is deleted due to the fusion of BCR and ABL1 genes, resulting in the breaking of this self-inhibition balance and the continuous activation of tyrosine kinase. ATP-competitive inhibitors targeting ABL1 have greatly improved the prognosis of CML patients, but drug resistance (Devos et al., 2021), disease progression (Kakiuchi et al., 2021), or some serious adverse events occurring during treatment bring challenges to clinical practices (Castagnetti et al., 2021; Clapper et al., 2021), especially drug resistance caused by BCR::ABL1 mutations (Mian et al., 2021). In the construction of cell lines carrying BCR::ABL1 mutants, we selected 1–2 common mutant sites in four regions including the ATP-binding loop (248–256 amino acids), TKI-binding site, catalytic domain (350–363 amino acids), and activation loop, which are the E255, T315, F359, L387, F486, and including T315 compound mutations. Imatinib, dasatinib, and ponatinib showed the weakened effect of growth inhibition and promoted apoptosis in cells with BCR::ABL1 compound mutations, which further verified the limitations of existing ATP-competitive TKIs targeting ABL1 for the treatment of CML patients with BCR::ABL1 compound mutations. ABL001, also named asciminib, targets the myristoyl pocket of ABL1 in an allosteric manner and simulates the natural N-terminal myristoyl peptide of ABL1, which restores the self-inhibitory conformation of tyrosine kinase and realizes the treatment effect of CML (Jones et al., 2020). ABL001 is effective for the treatment of CML patients with BCR::ABL1 single mutations but has a limited effect on those with BCR::ABL1 compound mutations (Eide et al., 2019), which has been further verified at the cellular level in this study.

GMB-475 is composed of an ABL1-binding element, a ligand targeting the E3 ligase Von Hippel–Lindau (VHL), and an intermediate linker. The BCR::ABL1-binding element binds to the target protein BCR::ABL1; the ligand targeting VHL recruits the E3 ubiquitin ligase VHL, and the intermediate linker “pulls the BCR::ABL1 protein closer to the E3 ubiquitin ligase,” resulting in the degradation of the BCR::ABL1 protein realized by the ubiquitin–proteasome system (Burslem et al., 2019). GMB-475 has unique advantages even if its binding mode is similar to ABL001. ATP-competitive TKIs and ABL001 inhibit the target protein by occupying the key site of ABL1, that is, the occupancy-driven pharmacological mode of traditional small molecule inhibitors, while PROTAC molecules including GMB-475, adopt the event-driven mode that is relatively less stringent for drug-binding sites, have the opportunity to realize degradation as long as they can bind to the target protein briefly, so that they have better compatibility with the target protein carrying mutations (Pettersson and Crews 2019; Xia et al., 2019; Kastl et al., 2021). We found that the IC50 value of ABL001 to Ba/F3-MIG-p210 cells carrying BCR::ABL1^T315I+F486S^ mutations was more than double that of GMB-475. GMB-475 can be recycled in theory after degrading BCR::ABL1, so its action time is longer than that of ABL1 inhibitors. However, GMB-475 showed significant effects of growth inhibition and promoted apoptosis in CML cell lines carrying BCR::ABL1 mutants only at high drug concentrations and performed a poor treatment effect on the CML mouse model. Combination therapy is an effective strategy for drug resistance. Afterward, we found that GMB-475 combined with dasatinib synergistically inhibited growth, promoted apoptosis, and blocked the cell cycle of Ba/F3 cells carrying BCR::ABL1 mutants, which reduced the effective concentration of the two drugs. For patients treated with dasatinib, the plasma concentration of dasatinib is relevant to efficacy and tolerability outcomes (García-Ferrer et al., 2019). It was recommended that the maximum plasma concentration of dasatinib was greater than 50 ng/ml to achieve clinical efficiency and that the plasma trough concentration was lower than 2.5 ng/ml to avoid adverse events such as pleural effusion (Miura 2015). The combination of GMB-475 and dasatinib can improve the therapeutic effect of dasatinib and may reduce its adverse effects. Due to the similar effects of ABL001 and GMB-475 on the ABL1 kinase and combination therapies of ABL001 with different TKIs being studied in the clinical setting already, we compared the synergistic effects between GMB-475 and ABL001 when in combination with dasatinib. The results indicated that GMB-475 combined with dasatinib showed more significant growth inhibition on Ba/F3-MIG-p210 cells than ABL001 combined with dasatinib, and the apoptosis rate of Ba/F3-MIG-p210 cells treated with GMB-475 plus dasatinib was higher than that treated with ABL001 plus dasatinib whether the drugs continued to act or were removed. GMB-475 combined with dasatinib exhibited a better synergistic effect compared with ABL001 combined with dasatinib. In addition to our study, there have been many studies concerning combination therapy to overcome TKI resistance, such as imatinib combined with farnesyl transferase inhibitors (Radujkovic et al., 2006) or mTOR inhibitors (Alves et al., 2019), dasatinib combined with decitabine (Abaza et al., 2020) or interferon-α2b (Hjorth-Hansen et al., 2016), and ABL001 combined with ponatinib (Gleixner et al., 2021), which is probably the one we are most interested in but did not provide a specific combination index, resulting in losing the opportunity to compare the synergistic effect with this study.

We detected the changes in intracellular signaling pathways induced by GMB-475, dasatinib, and GMB-475 plus dasatinib to explore the synergistic mechanism. Due to the inconsistent sensitivity of different BCR::ABL1 mutations to GMB-475 (the IC50 values of GMB-475 to Ba/F3-MIG-p210 cells with BCR::ABL1^T315I^, BCR::ABL1^T315I+E255K^, BCR::ABL1 ^T315I+L387M^, and BCR::ABL1^T315I+F486S^ at 48 h were 3.69, 8.29, 3.70, and 4.49 µM, respectively), we used distinct treatment concentrations for different mutations in order to avoid the influence of too many dead cells on the experimental results. GMB-475 and dasatinib synergistically downregulated the expression of BCR::ABL1 and some proteins of the JAK-STAT axis in Ba/F3 cells carrying BCR::ABL1 mutants, such as p-JAK2, p-STAT5a, STAT3, MYC, and Bcl2. The JAK-STAT pathway is widely involved in important biological processes, such as cell proliferation, differentiation, apoptosis, and immune regulation (Xin et al., 2020), and GMB-475 combined with dasatinib synergistically regulated the expression levels of some genes in this pathway, which may be the significant mechanism for the synergistic antitumor effect of the two drugs.

In conclusion, the combination of PROTAC molecules targeting ABL1 in an allosteric manner and ATP-competitive TKIs provides a novel idea for the treatment of CML patients with highly resistant BCR::ABL1 mutations in clinical practice. GMB-475 as a single therapy may have great limitations; however, combination therapy based on that has a potential treatment value for CML patients, but further clinical studies are needed for verification.

## Data Availability

The original contributions presented in the study are included in the article/Supplementary Material; further inquiries can be directed to the corresponding author.

## Abbreviations

CML, chronic myeloid leukemia; PROTAC, proteolysis targeting chimera; DAS, dasatinib; WT, wild type; TKIs, tyrosine kinase inhibitors; MTT, thiazolyl blue tetrazolium bromide; CCK8, cell counting kit-8; CI, combination index; qPCR, quantitative real-time PCR; CTR, control.
