# Supplementary material for: The proteolysis targeting chimera GMB-475 combined with dasatinib for the treatment of chronic myeloid leukemia with BCR::ABL1 mutants
Source: Front Pharmacol. 2022 Oct 3;13:931772. doi: 10.3389/fphar.2022.931772 (PMC9574342; doi:10.3389/fphar.2022.931772)
Supplement: Supplementary file 1 [file DataSheet1.pdf]

A

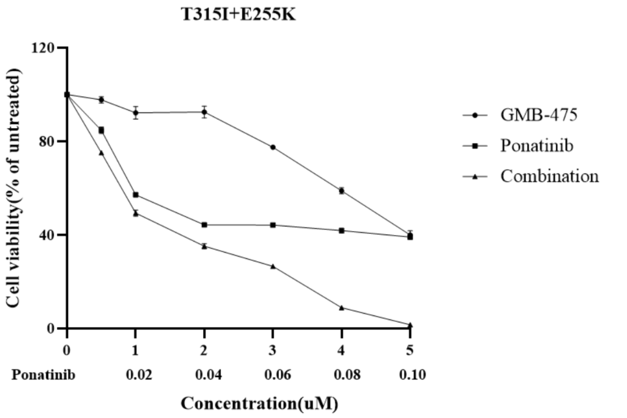

B

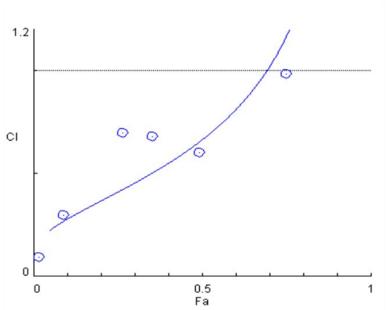

GMB-475	Ponatinib	Effect	CI
0.5	0.010	0.7515	0.98364
1.0	0.020	0.4931	0.60417
2.0	0.040	0.3519	0.68113
3.0	0.060	0.2655	0.69566
4.0	0.080	0.0892	0.29630
5.0	0.100	0.0159	0.09481

ED50	ED75	ED90	ED95
0.66583	1.17309	2.19673	3.44577

C

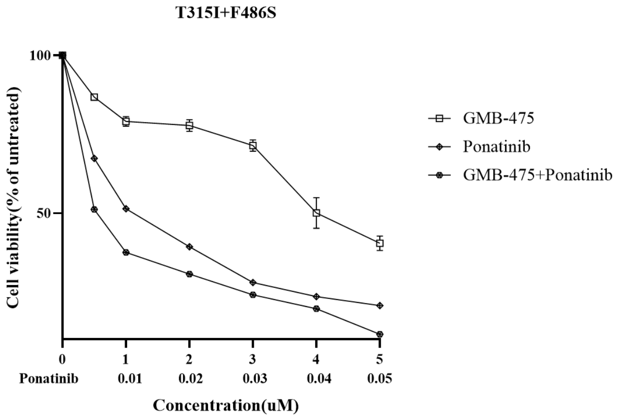

D

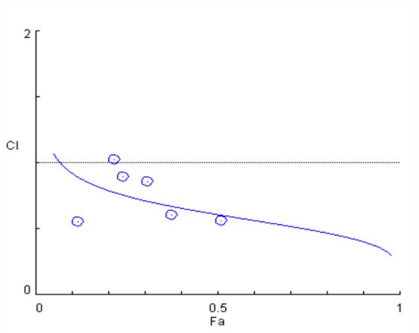

GMB-475	Ponatinib	Effect	CI
0.5	0.005	0.5116	0.56129
1.0	0.010	0.3754	0.60521
2.0	0.020	0.3069	0.86173
3.0	0.030	0.2410	0.89299
4.0	0.040	0.2170	1.02350
5.0	0.050	0.1161	0.55770

ED50	ED75	ED90	ED95
0.60812	0.49438	0.40195	0.34918

Supplement Figure 1 GMB-475 combined with ponatinib had synergistic effects on the growth inhibition of Ba/F3-MIG-p210 cells with BCR-ABL1 mutants. (A and C) The cell viability of Ba/F3-MIG-p210 cells carrying BCR-ABL1$^{T315I+E255K}$ or BCR-ABL1 $^{T315I+F486S}$ mutations treated with different concentrations of GMB-475, ponatinib or GMB-475 plus ponatinib for 48 hours was detected by CCK8 assay. The abscissa represented the concentrations of GMB-475, and the corresponding concentrations of ponatinib were marked below the abscissa; the ordinate was the cell survival rate. (B and D) The curve figures of combination indexes (CIs); the CIs of GMB-475 combined with ponatinib at ED50, ED75, ED90 and ED95, or the CIs for different concentrations of GMB-475 combined with ponatinib were shown below the figures; the CI at ED50 was the overall CI.

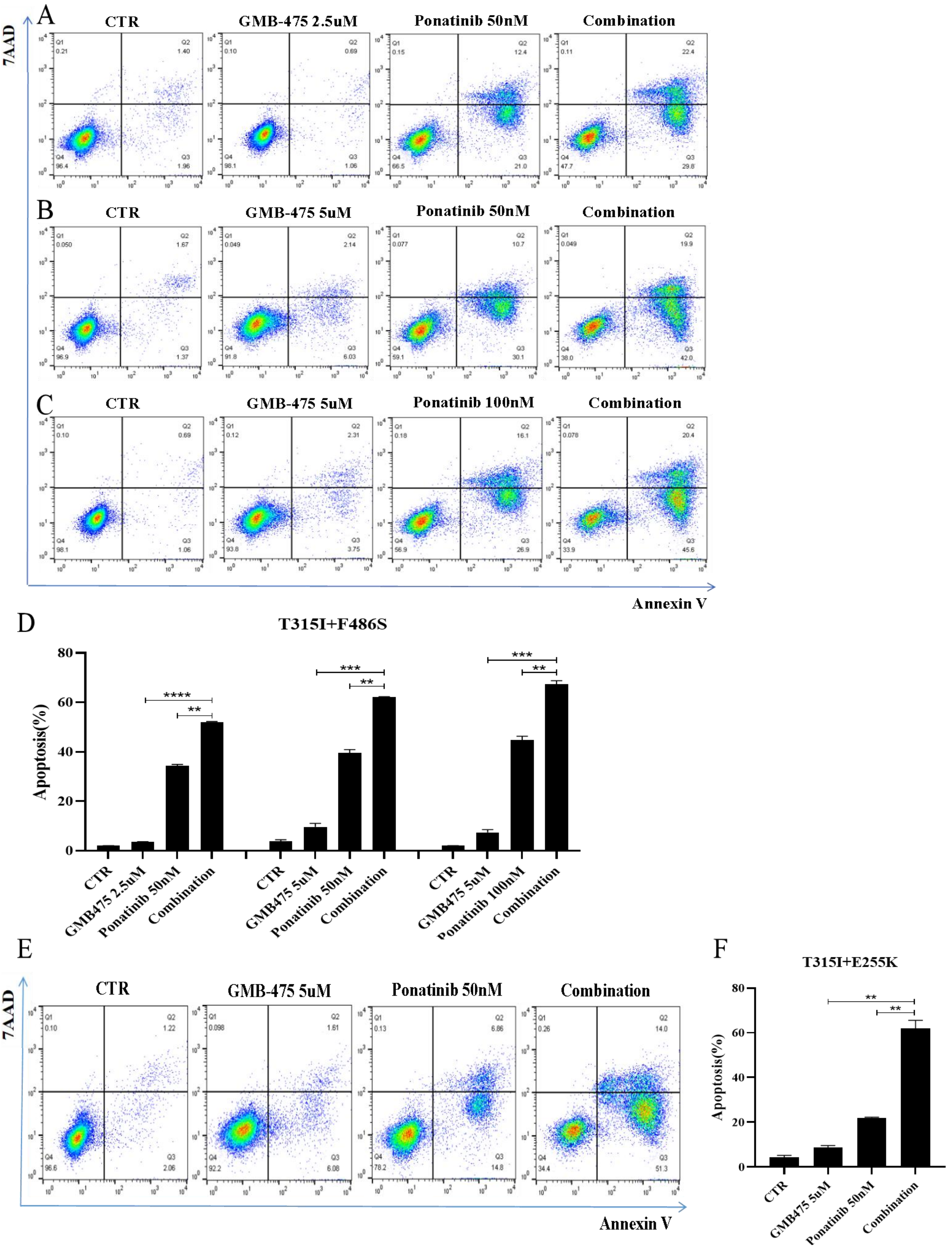

Supplement Figure 2 GMB-475 combined with ponatinib synergistically promoted the apoptosis of Ba/F3-MIG-p210 cells. The apoptosis of Ba/F3-MIG-p210 cells treated with control medium (CTR), GMB-475, ponatinib or GMB-475 plus ponatinib for 48 hours was detected by Annexin V and 7AAD double staining: (A-C) Ba/F3-MIG-p210 cells carrying BCR-ABL1$^{T315I+F486S}$ mutations were treated with CTR, ponatinib (50nM or 100nM), GMB-475 (2.5uM or 5uM) and GMB-475 plus ponatinib respectively. (D) Statistical analysis results of figures A-C. (E) Ba/F3-MIG-p210 cells carrying

BCR-ABL1$^{T315I+E255K}$ mutations were treated with CTR, ponatinib (50nM), GMB-475 (5uM) and GMB-475 plus ponatinib respectively. (F) Statistical analysis results of figure E.

Supplement Table 1 Main reagents and suppliers

Reagents	Suppliers
IL-3	R&D Systems
RPMI 1640 medium	Corning
Fetal Bovine Serum	GEMINI
Rabit anti- C-Abl, p-AKT (S473), pan-AKT, p-STAT5a (Y694), and c-MYC antibodies	CST
Rabit anti- JAK2, p-JAK2 (Y1007+Y1008), JAK2, STAT5a, STAT3, and Bcl2 antibodies	HUABIO
Rabit anti-β-actin antibody	EnoGene
sheep anti-rabbit IgG-HRP	BioX
RIPA Lysis Buffer (medium)	Beyotime Biotechnology
BCA Protein Assay Kit	ComWin Biotech
5×SDS loading buffer	Solarbio
Prestained protein marker	Epizyme
PVDF membrane	Millipore
Immobilon Western HRP substrate	Millipore
GMB-475	Selleck / Targetmol
Dasatinib, imatinib	Sigma-Aldrich
Ponatinib	Targetmol
ABL001	Selleck
MTT	Solarbio
Cell Counting Kit-8（CCK8）	UE
Annexin V-APC	UE
7AAD	AAT Bioquest
Cell cycle assay kit	Sungene Biotech
RNA extraction kit	Fuji Biotechnology
Reverse transcription kit	Takara
2x SYBR Green qPCR Master Mix	Bimake
Phosphate buffered saline	ZSGB-Bio
Blasticidin (BSD) (10mg/mL)	BioX
D-PBS	Thermo-Gibco™
D-luciferin potassium salt	Meilunbio
HBLV-LUC-BSD (LV47092512) virus	HANBIO

Supplement Table 2 The primer sequences of genes in qPCR

Genes	Forward primer	Reverse primer
JAK2	GTGTCGCCGGTTCCTGA	TTGTAAGGCAGGCCATTCCC
STAT5a	ACTCCTGTACTTGGTTCGTCA	CCAGGTCAAACTCGCCATCT
MYC	GATTCCACGGCCTTCTCTCC	TTCTTGCTCTTCTTCAGAGTCG
Bcl2	GGATAACGGAGGCTGGGATGC	ACTTGTGGCCCAGGTATGC
AKT1	GCCGCCTGATCAAGTTCTCC	TTCAGATGATCCATGCGGGG
STAT3	CCCCGTACCTGAAGACCAAG	TCCTCACATGGGGGAGGTAG
mTOR	ACCAAAGGAAATGCAGAAGCC	CATCTCTTCTCTCAGACGCTCTCC
GAPDH	GGGTCCCAGCTTAGGTTCATC	CCAATACGGCCAAATCCGTTC
